# Supplementary material for: 1Hz vs. cTBS Transcranial Magnetic Stimulation to Augment Behavior Therapy for Tics: A Randomized Clinical Trial
Source: medRxiv. 2026 Oct 1:2026.09.29.26364036. Preprint. [Version 1] doi: 10.64898/2026.09.29.26364036 (PMC13648794; doi:10.64898/2026.09.29.26364036)
Supplement: Supplementary file 1 [file media-1.docx]

**Consort diagram: Phase 1 of the CBIT+TMS trial**

**Additional information about non-completers:** Non-completers (n = 2) included one cTBS participant who withdrew after session 1 due to disinterest in CBIT and another in sham who was withdrawn from TMS due to a status change that impacted their risk profile; this participant completed CBIT and all assessments except for the post-MRI.
